# The first complete mitochondrial genome of *Actinopyga* from *Actinopyga echinites* (Aspidochirotida: Holothuriidae)

**DOI:** 10.1080/23802359.2019.1710598

**Published:** 2020-01-27

**Authors:** Shengping Zhong, Lianghua Huang, Yonghong Liu, Guoqiang Huang

**Affiliations:** a Institute of Marine Drugs, Guangxi University of Chinese Medicine, Nanning, China;; b Key Laboratory of Marine Biotechnology, Guangxi Institute of Oceanology, Beihai, China

**Keywords:** Mitochondrial genome, *Actinopyga echinites*, Holothuroidea

## Abstract

The deep-water redfish, *Actinopyga echinites*, is an ecologically and economically important holothuroid in China due to its valuable nutrition and pharmacological compounds. However, the taxonomy and phylogeny of the *Actinopyga* have been debated and misidentifications have been reported recently. Moreover, there remain considerable doubts about cryptic species complex within *Actinopyga*. In this study, we report the first complete mitochondrial genome of *Actinopyga* from *A. echinites*. The mitogenome has 15,619 base pairs (62.9% A + T content) and made up of a total of 37 genes (13 protein-coding, 22 transfer RNAs, and 2 ribosomal RNAs), and a putative control region. This study was the first available complete mitogenome of *Actinopyga* and will provide useful genetic information for future phylogenetic and taxonomic classification of Holothuriidae.

Holothuroids, or sea cucumbers (Holothuroidea) are a diverse group of echinoderms and it contains about 1400 valid species around the world in 6 orders and 25 families, which are the most valuable and vulnerable inshore fisheries resources (Miller et al. 2017). *Actinopyga* is one of the five genera in the family Holothuriidae, which contains about 16 valid species (Samyn et al. 2006). However, due to limited molecular systematic studies and morphological environment plasticity, the taxonomy and phylogeny of the *Actinopyga* have been debated and misidentifications have been reported recently (Samyn et al. 2006; Miller et al. 2017). The deep-water redfish *Actinopyga echinites*, widely distributed throughout the tropical Indo-west Pacific region including the Red Sea, is an ecologically and economically important species in China which was known as a delicious seafood and a traditional medicine for its pharmacological compounds (Melek et al. 2012). The complete mitochondrial genome is an excellent molecular marker for studying phylogenetic relationships and species identification. Here, we first completed the mitochondrial genome of *Actinopyga* from *A. echinites*, which provided useful genetic markers for species identification and taxonomy assessment.

Tissue samples of *A. echinites* from three individuals were collected from HaiNan province, China (SanYa, 18.225361 N, 109.379574 E), and the whole-body specimen (#GH0037) were deposited at Marine biological Herbarium, Guangxi Institute of Oceanology, Beihai, China. The total genomic DNA was extracted from the muscle of the specimens using an SQ Tissue DNA Kit (OMEGA, Guangzhou, China) following the manufacturer’s protocol. DNA libraries (350 bp insert) were constructed with the TruSeq NanoTM kit (Illumina, San Diego, CA) and were sequenced (2 × 150 bp paired-end) using HiSeq platform at Novogene Company, China. Mitogenome assembly was performed by MITObim (Hahn et al. 2013). The cytochrome oxidase subunit I (COI) gene of *A. echinites* (GenBank accession number: FJ589209) was chosen as the initial reference sequence for MITObim assembly. Gene annotation was performed by MITOS (http://mitos2.bioinf.uni-leipzig.de).

The complete mitogenome of *A. echinites* was 15,619 bp in length (GenBank accession number: MN793975), containing the typical set of 13 protein-coding, 22 tRNA, and 2 rRNA genes, and a putative control region. The overall base composition of the mitogenome was estimated to be A 34.0%, T 28.9%, C 20.9% and G 16.1%, with a high A + T content of 62.9%, which is similar, but higher than *Holothuria leucospilota* (57.6%) (Zhong et al. 2019). The mitogenomic phylogenetic analyses showed that *A. echinites* was first clustered with *H. forskali* in the monophyletic Holothuriidae clade (Figure 1), which is consistent with the phylogenetic analyses of family Holothuriidae using 16S mitochondrial ribosomal DNA (Kerr et al. 2005). Our mitogenome data supported the close relationship between genus *Actinopyga* and *Holothuria*. The complete mitochondrial genome sequence of *A. echinites* was the first sequenced mitogenome in *Actinopyga*, which will be useful for its taxonomy research and future conservation and management.

**Figure 1.**
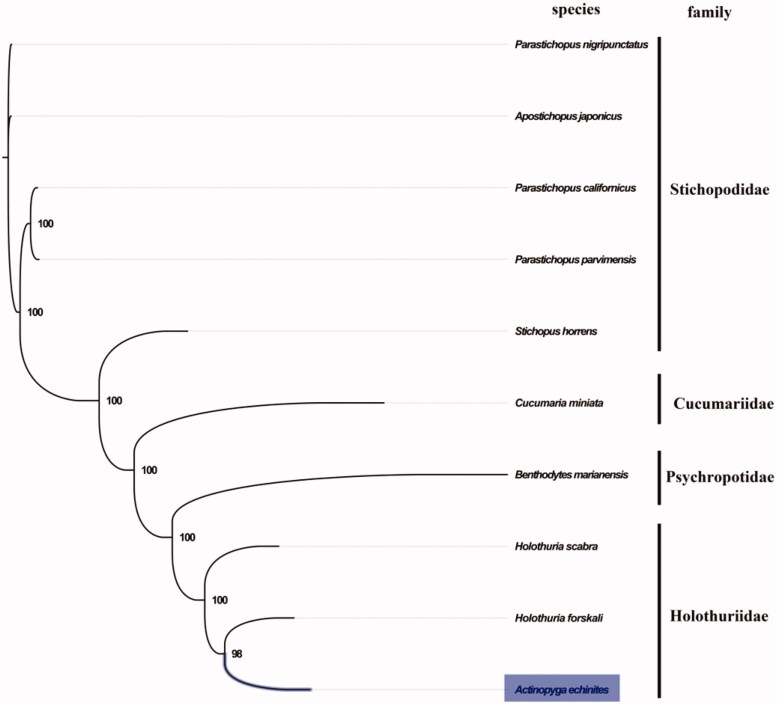
Phylogenetic tree of 10 species in Holothuroidea. The complete mitogenomes are downloaded from GenBank and the phylogenic tree is constructed by the maximum-likelihood method with 100 bootstrap replicates. The bootstrap values were labeled at each branch node. The gene accession number for tree construction is listed as follows: *Parastichopus nigripunctatus* (NC_013432), *Apostichopus japonicus* (NC_012616), *Parastichopus californicus* (NC_026727), *Parastichopus parvimensis* (NC_029699), *Stichopus horrens* (NC_014454), *Cucumaria miniata* (NC_005929), *Benthodytes marianensis* (NC_040968), *Holothuria scabra* (NC_027086), and *Holothuria forskali* (NC_013884).
